# The alleviating effect of *Scutellaria amoena* extract on the regulation of gut microbiota and its metabolites in NASH rats by inhibiting the NLRP3/ASC/caspase-1 axis

**DOI:** 10.3389/fphar.2023.1143785

**Published:** 2023-11-07

**Authors:** Yu-Ping Lin, Qiong-Lian Fang, Sheng-Nan Fu, Xin-Ping Li, Rui Shi, Cheng-Hong Du, Xue Qiao, Xun-Qing Yin, Yong-Cheng Zeng, Xiu-Juan Zhao, Yan Hua

**Affiliations:** ^1^ Key Laboratory for Forest Resources Conservation and Use in the Southwest Mountains of China, Southwest Forestry University, Kunming, China; ^2^ School of Chinese Materia Medica, Yunnan University of Chinese Medicine, Kunming, China

**Keywords:** Scutellaria amoena, NASH, NLRP3/ASC/caspase-1 axis, gut microbiota, metabolites

## Abstract

**Background:** *Scutellaria amoena* (SA) is the root of *S. amoena* C.H. Wright of Labiatae, also known as *Scutellaria* southwestern. This is mainly distributed in Sichuan, Yunnan, and Guizhou in China. In southwest China, SA is used as an alternative method to genuine medicine for the treatment of allergy, diarrhea, inflammation, hepatitis, and bronchitis. Thus far, studies on the effects of SA on non-alcoholic steatohepatitis (NASH) are lacking. This paper investigated the effect of SA on the regulation of gut microbiota and its metabolites in NASH rats by inhibiting the NOD-like receptor 3 (NLRP3)/apoptosis-associated speck-like protein (ASC)/caspase-1 axis.

**Methods:** A NASH rat model was induced by a high-fat diet (HFD) for 12 weeks, and rats were orally given different doses of SA extracts (150 and 300 mg/kg/d) for 6 weeks. Changes in histological parameters, body weight, organ indexes, cytokines, and biochemical parameters related to NLRP3 in NASH rats were checked. 16S rRNA gene sequencing and UPLC-MS/MS technology were used to analyze the changes in the gut microbiota composition and its metabolites in NASH rats.

**Results:** SA significantly inhibited the HFD-induced increase in body weight, lipid levels, and inflammatory infiltration. SA notably inhibited the HFD-induced increase in the upper and lower factors of NLRP3, such as transforming growth factor (TGF)-β, tumor necrosis factor (TNF)-α, interleukin (IL)-6, IL-18, pro-IL-18, IL-1β, pro-IL-1β, NLRP3, ASC, and caspase-1. Additionally, mRNA expressions of caspase-1, NLRP3, and ASC were significantly downregulated after SA treatment. The results of the intestinal flora showed that SA could increase the diversity of flora and change its structure and composition in NASH rats by reducing Firmicutes/Bacteroidetes (F/B) ratio, *Blautia* (genus), Lachospiraceae (family), and Christensenellaceae *R-7 group* (genus), and increasing Muribaculaceae (family) and *Bacteroides* (genus). The metabolomics revealed that 24 metabolites were possibly the key metabolites for SA to regulate the metabolic balance of NASH rats, including chenodeoxycholic acid, xanthine, and 9-OxoODE. Nine metabolic pathways were identified, including primary bile acid biosynthesis, bile secretion, purine metabolism, and secondary bile acid biosynthesis.

**Conclusion:** SA can regulate the intestinal microbial balance and metabolic disorder by inhibiting the NLRP3/ASC/caspase-1 axis to relieve NASH.

## Graphical Abstract

**Figure:**
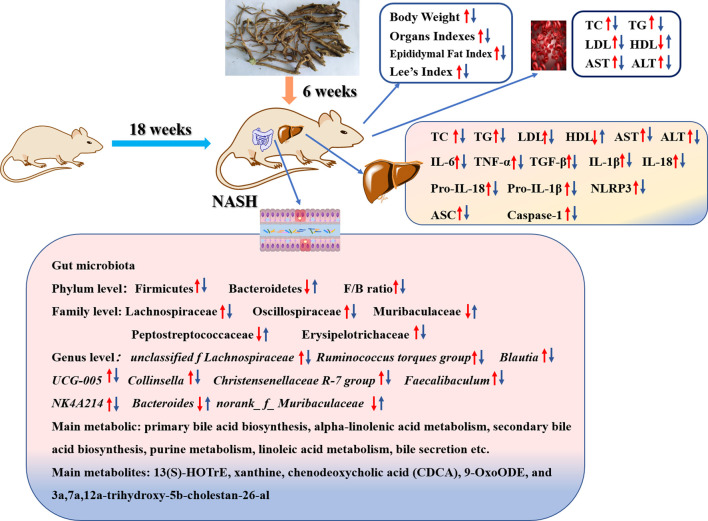
*Scutellaria amoena* extract regulates HFD-induced gut microbial disorder and changes the level of the flora metabolism and its metabolites in NASH rats, thereby reducing inflammation levels, improving lipid metabolism, and inhibiting NLRP3/ASC/caspase-1 axis dysfunction.

## Introduction

Non-alcoholic fatty liver disease (NAFLD) is a common chronic liver disease associated with a high metabolic risk of health problems, such as obesity, insulin resistance, dyslipidemia, and type 2 diabetes (T2D). Non-alcoholic steatohepatitis (NASH) is a more severe form among three typical pathological subtypes of NALFD (Xu et al., 2020). It is a progressive disorder characterized by excessive hepatic steatosis, balloon-like changes in morphology, and diffuse hepatic lobular mild inflammation, and will lead to cirrhosis, liver fibrosis, and cancer (Benedict and Zhang, 2017; Powell et al., 2021). Previous studies have shown that the incidence of NASH is 10%–20% in the general population of Western countries and 60% in patients with obesity and T2D (Mridha et al., 2017). In recent years, improvements in living standards, changes in dietary structure, and drug abuse have led to an increase in the incidence of NASH, while with increases in childhood obesity, the age of NASH onset has been lowered (Oliveira et al., 2018). NASH can progress to non-alcoholic fatty liver fibrosis, cirrhosis, and primary hepatocellular carcinoma (HCC). However, no drug is currently approved for the treatment of NASH (Roeb and Geier, 2019; Raza et al., 2021).

Recently, the NOD-like receptor (NLR) NLRP3 inflammasome was shown to play a key role in the progress of NASH (Huang et al., 2021). When the NLRP3 receptor, pro-caspase-1, and ASC adapter interacted, the assembly of NLRP3 inflammasome finished as a platform of caspase-1 activation (Hou et al., 2020; Yu et al., 2022). Activated caspase-1 mediates the cleavage of inflammatory cytokines pro-IL-18 and pro-IL-1β into mature forms of IL-18 and IL-1β and triggers an inflammatory response (Dong et al., 2020; Knorr et al., 2020). Therefore, IL-1β and IL-18 secretion depend on the NLRP3/ASC/caspase-1 axis (Zhang et al., 2019). Lipid metabolism disorders often occur in HFD-induced NASH rats, such as abnormalities in triglyceride (TG), low-density lipoprotein cholesterol (LDL-C), total cholesterol (TC), and high-density lipoprotein cholesterol (HDL-C) levels (Chen et al., 2018). Lipid peroxidation increases the levels of certain inflammatory factors, such as IL-6, TNF-α, IL-1β, and TGF-β, and these inflammatory factors, in turn, activate NLRP3 and form a circulating inflammatory storm (Fang et al., 2022). In summary, NLRP3 inflammasome may play a critical role in the prevention and treatment of NASH.

**FIGURE 1:**
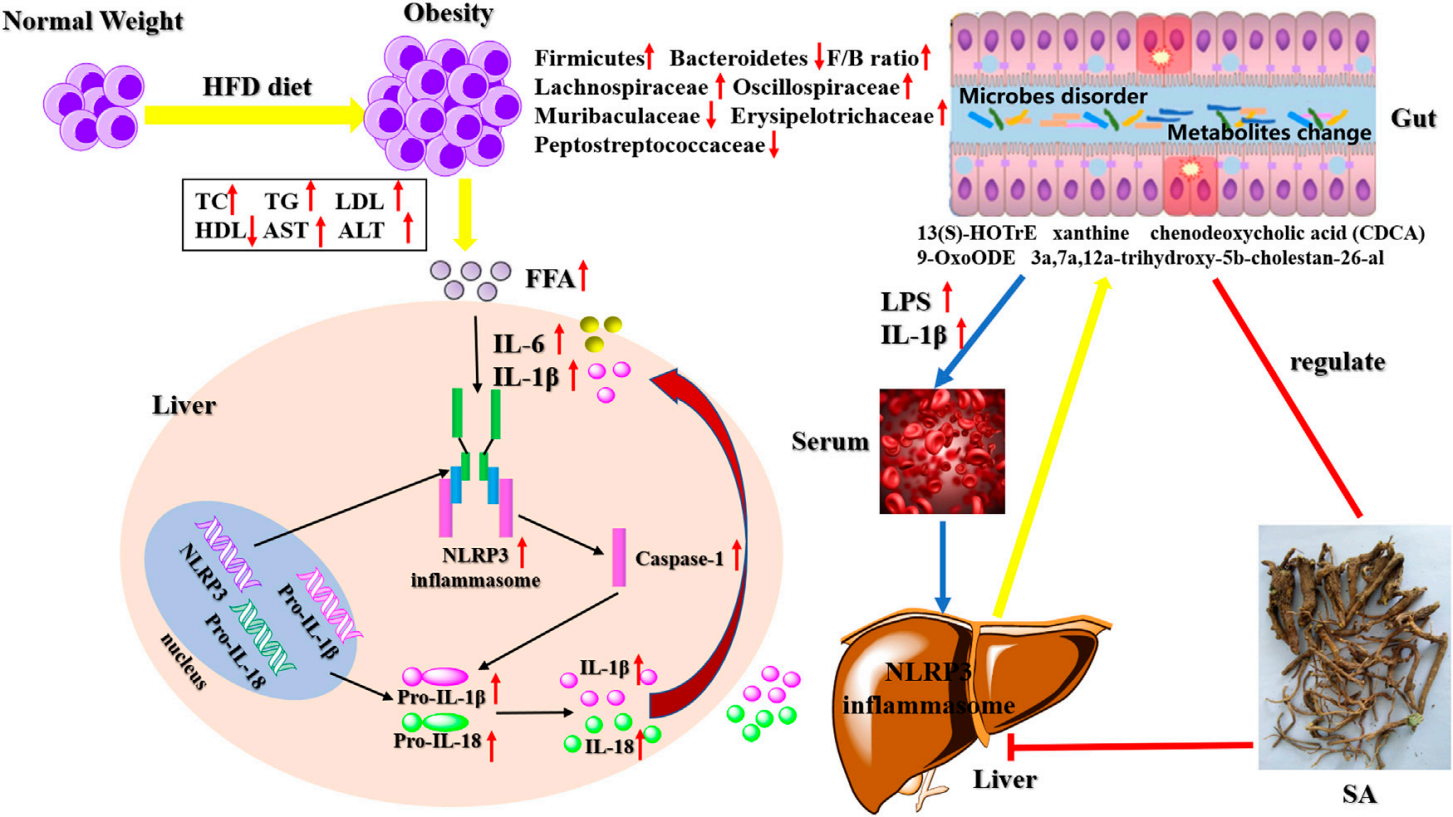
Activation mechanisms of Non-Canonical and Alternative NLRP3 inflammasome pathways and the resulting intestinal flora disorders and metabolites change.

Some studies have found that NLRP3 inflammasome can recognize intestinal bacteria and their metabolites and perform the intestinal immune function, thereby protecting the intestinal tract (Ding et al., 2019; Liao et al., 2019). The gut microbiome provides an environmental factor for the development and progression of NAFLD-HCC (Zhang et al., 2021). Ji et al. (2019) found an increase in the abundance of Firmicutes (the predominant Gram-positive bacterium) in mice fed with an HFD, and this change was associated with an increase in peptidoglycan (PGN), lipopolysaccharide (LPS), and lipoproteins. Compared with steatosis, the abundance of *Bacteroides* in intestinal microflora related to NASH is higher than that in the intestinal microflora related to fibrosis (Chen and Vitetta, 2020). After administering HFD, the changes in the consist of these microorganisms led to the disorder of their metabolites, such as bile acids, short-chain fatty acids (SCFAs), trimethylamine, and ethanol, and the signaling pathways that they affect may contribute to the development of NAFLD (Albillos et al., 2020; Aron-Wisnewsky et al., 2020). Additionally, the intestinal microflora is very important for maintaining tissue homeostasis and intestinal permeability, which is increased in NAFLD/NASH patients, while some inflammatory factors, such as IL-1β, TNF-α, and IL-6 easily penetrate the external intestinal tissues, causing inflammation. The observed inflammatory changes may be caused by the increase in intestinal permeability, especially in NASH patients (Kolodziejczyk et al., 2019). Activation mechanisms of Non-Canonical and Alternative NLRP3 inflammasome pathways and the resulting intestinal flora disorders and metabolites change (Figure 1).

Silybin (SB) has anti-inflammatory, antioxidant, and antifibrotic capabilities and is used for different liver diseases, especially chronic liver disease, liver cirrhosis, and HCC (Federico et al., 2017). Therefore, it was selected as a positive drug. However, it has low solubility and poor bioavailability. *Scutellaria amoena* (SA), first recorded in “Diannan Bencao” and “Ancient Books of Yi Medicine,” has been used as traditional Chinese and ethical medicine for over 2,000 years (Fu et al., 2018). SA is mainly distributed in the provinces of Yunnan, Sichuan, and Guizhou in China (Li et al., 2022). It has been reported that SA mainly comprises flavonoids and has antibacterial, antiviral, antioxidant, antineoplastic, and protective cardiovascular functions (He et al., 2013; Liu et al., 2016). Additionally, our previous study indicated that total flavonoids from SA have a protective effect on CCl_4_-induced liver injury (Fu et al., 2020). However, no studies on the function of SA in NASH by regulating NLRP3/ASC/caspase-1 axis have been reported until now. In this study, we investigated the alleviating effect and the underlying mechanism of SA in HFD-induced NASH. The results indicated that SA reduced HFD-induced NASH by inhibiting NLRP3 through the regulation of intestinal flora and its metabolites. In summary, SA may be a promising drug to treat NASH.

## Materials and methods

### Materials

SB capsules were obtained from Tianjin Tianshili Shengte Pharmaceutical Co., Ltd. (China). Alanine aminotransferase (ALT), aspartate aminotransferase (AST), TG, TC, LDL-C, and HDL-C were acquired from Nanjing Jiancheng Bioengineering Institute (China, No: 20220328). IL-6, TNF-α, pro-IL-1β, IL-18, TGF-β, IL-1β, pro-IL-18, NLRP3, caspase-1, and ASC commercial kits were acquired from JiangSu Meimian Industrial Co. Ltd. (China, No: 20032603R, 20032608R, 220427047R, 220427045R, 20032602R, 20032606R, 220427042R, 20032618R, 20032620R, and 20032623R). Hematoxylin and eosin (H&E) stain was obtained from Wuhan Google Biotechnology Co., Ltd. (China). 4% paraformaldehyde was acquired from Beijing Labgic Technology Co., Ltd. (China), and Multistrix SG Paraffin wax (56°C–58°C) was obtained from Wuhan Seville Biotechnology Co., Ltd. (China). Rat anti-ASC and rat anti-caspase-1 were acquired from Beijing Biosynthesis Biotechnology Co., Ltd. (China, No: BB01251573 and BJ10091395), and rat anti-NLRP3 was obtained from Abcam Plc (United Kingdom, No: EPR23094-1). The basic feed (high-gluten flour 4.1%, primary secondary powder 29%, corn 40.84%, fish meal 2%, and soybean oil 2%, 46 soybean meal 16.06%) was purchased from SPF (Beijing) BIOTECHNOLOGY Co., Ltd. (China), and HFD (comprised of 10% refined lard, 82.5% basic diet, 2% cholesterol, 0.5% sodium cholate, and 5% egg yolk) was made by our research group.

### Preparation of SA

The medicinal materials of SA were gathered from Xinping County in Yunnan Province (China) and identified by Professor Chun-Xia Pu (Yunnan University of Chinese Medicine). The voucher specimens (L20170803) were deposited at Pharmaceutical and Food Resources Development Laboratory, School of Chinese Materia Medica, Yunnan University of Chinese Medicine, Kunming, China. In the extraction method, 1 kg of dried root powder of SA was weighed and extracted using reflux three times with 95% ethanol for 2 h. Filtered and combined that extract and removed from the solvent to obtain SA. The SA extract was freeze-dried under reduced pressure and stored at 4°C, and the freeze-dried powder of SA total extract (1 g) was equivalent to 3.6 g of the raw materials of SA. Its ingredients are mainly flavonoids, including tenaxin Ⅱ-7-O-β-D- glucuronopyranoside, baicalin, chrysin-7-O-β-D-glucurunopyranoside, oroxylin A-7-O-β-D-glucurunopyranoside, norwogonin-7-O-β-D-glucopyranoside 6″-methyl ester, norwogonin, baicalein, wogonin, chrysin, and oroxylin A, and the content of baicalin was 0.06 mg/g (Fang et al., 2023).

### Animal and experimental design

Thirty specific pathogen-free (SPF) SD rats (male, 6–8 weeks, weight, 200 ± 20 g) were purchased from Hunan Slack Jingda Experimental Animal Co., Ltd., China [permission number: SCXK (Hunan) 2016-0002]. All animals were housed under the conditions at 22°C ± 3°C, 55% ± 5% humidity, and a 12-h light/dark cycle. According to the guidelines of the National Science and Technology Committee of China, animals were provided with free food (standard commercial rat food) and water. After 1 week of adaptive feeding, rats were randomized into the following five groups (*n* = 6 rats per group): 1) normal control (CON) group (normal saline); 2) HFD model (MOD) group (normal saline); 3) Sylibin (SB) group (12.6 mg/kg/d); 4) high-dose SA (SA-H) group (300 mg/kg/d); 5) low-dose SA (SA-L) group (150 mg/kg/d). The corresponding test drug (normal saline) was intragastrically administered to rats once daily for 6 consecutive weeks. SB and SA were separately prepared in normal saline. NASH was HFD-induced for 18 consecutive weeks in all groups except for the CON group. Throughout the experiment, all animals were weighed weekly to calculate their body weight (BW), and the food intake of all rats was recorded daily.

### Sample collection

Experiments were completed after 18 weeks, all animals fasted for 12 h and then were anesthetized with 10% pentobarbital sodium. The final BW and body lengths were measured for calculating Lee’s index ( ${\text{Lee}}^{\prime }s\text{ index}=\text{body weight }\left[g\right]/\text{body length }\left[\text{cm}\right]\times 100$ ). After sacrificing the rats, blood samples were collected for serum (4°C, 3,500 rpm, 15 min). Fresh livers were harvested and weighed to calculate the liver index ( $\text{liver index }\left[\%\right]=\text{liver weight }\left[g\right]/\text{BW }\left[g\right]\times 100$ ). Some livers were fixed in 4% paraformaldehyde for paraffin sectioning, which was cut into 3–5-μm sections for H&E staining and immunohistochemistry (Servicebio, China). Other livers were homogenized in the ratio of normal saline and liver tissue (w: v = 1: 9) for enzyme-linked immunosorbent assay (ELISA). The cecum contents of the rats were collected and stored in a −80°C refrigerator for the subsequent analysis.

### Biochemical and ELISA analysis

The levels of AST, ALT, LDL-C, HDL-C, TC, and TG (Nanjing Jiancheng Bioengineering Institute, China) in serum and liver were determined on the INFINITUM 200 PRO plate reader (TECAN, Switzerland). The levels of IL-6, IL-1β, pro-IL-18, pro-IL-1β, IL-18, TGF-β, TNF-α, NLRP3, caspase-1, and ASC in the liver tissues of rats were quantified using ELISA kits (Jiangsu Meimian Industrial Co., Ltd., China), according to the manufacturer’s instructions.

### Liver histopathology and immunohistochemistry

The rat liver tissue was fixed with 4% paraformaldehyde stationary liquid, dehydrated, and made into wax sections. Tissue sections were stained with H&E and observed under an optical microscope for histopathological study. The paraffin sections were dewaxed and incubated in 3% hydrogen peroxide solution. Then, 3% bovine serum albumin (BSA) was added for closure for 30 min, and then the blocking fluid was removed, followed by adding the primary antibody for incubation. After the sections were dried, the secondary antibody [horseradish peroxidase (HRP)-labeled] was added, and the sections were measured using an optical microscope. All immunohistochemical analyses were repeated three times, and representative images were captured, presented, and quantified by a semi-quantification software ImageJ.

### RT-qPCR (real-time quantitative reverse transcription-polymerase chain reaction)

Total RNA was extracted from the liver tissues using TRIzol reagent (Cowin, Jiangsu, China). The extracted RNA was reverse transcribed into cDNA using SuperRT cDNA Synthesis Kit cDNA (Cowin). We used 2 μL of cDNA template for PCR amplification using the following primers: NLRP3 forward 5′-CAG​CGA​TCA​ACA​GGC​GAG​AC-3′, reverse 3′-AGA​GAT​ATC​CCA​GCA​AAC​CTA​TCC​A-5′; ASC 5′-TGT​GCT​TAG​AGA​CAT​GGG​CAT​ACA​G-3′, 3′-GCC​ATA​CAG​AGC​ATC​CAG​CAA-5′; caspase-1 5′-CCA​GAG​CAC​AAG​ACT​TCT​GAC-3′, 3′-TGG​TGT​TGA​AGA​GCA​GAA​AGC-5′; GAPDH 5′-GCC​CAG​CAA​GGA​TAC​TGA​GA-3′, 3′-GTA​TTC​GAG​AGA​AGG​GAG​GGC-5′. The RT-qPCR analysis was performed using the real-time PCR kit following the manufacturer’s instructions. The gene expression levels were calculated using the 2^−ΔΔCT^ method and normalized to that of *GAPDH*.

### Western blot analysis

Hepatic proteins were estimated by western immunoblotting, and antibody conditions were provided upon req of that manufacturer. Proteins were run on 10% SDS‐PAGE and transferred to polyvinylidene fluoride (PVDF) membranes. The blots were subsequently incubated with rat anti-ASC (1:1000), rat anti-caspase-1 (1:1000), rat anti-NLRP3 (1:1000), and rat anti-β-actin (1:2000) primary antibodies at 4°C overnight, followed by the incubation with HRP-conjugated secondary antibodies (1:2000) for 1 h and visualization.

### Gut microbiota analysis

The total genomic DNA was extracted from the rats’ cecum contents using the E.Z.N.A.^®^ soil DNA Kit (Omega Bio-Tek, Norcross, GA, United States) following the manufacturer’s instructions. The V3-V4 regions of the bacterial 16S rRNA gene were amplified with the primer 338F (5′-ACT​CCT​ACG​GGA​GGC​AGC​AG-3′); 806R (5′-GGACTACHVGGGTWTCTAAT-3′) using the ABI Gene Amp^®^ 9700 PCR thermocycler (Applied Biosystems, United States). Analysis was performed at the genus, family, and phylum levels.

The RDP (http://rdp.cme.msu.edu/) classifier algorithm was used to classify and annotate the species and compare them with the genes in the Sliva (Release 132 http://www.arb-silva.de) database. The 16S rRNA gene database with a confidence threshold of 70% was also used. Using I-sanger to evaluate microbial differences analysis and correlation analysis, all data assessments were performed on the Majorbio Cloud Platform (MajorbioBio-Pharm Technology Co., Ltd. Shanghai, China).

### Gut metabolites analysis

50 mg of cecal contents were accurately weighed and extracted with 400 μL of methanol: water (4:1, v/v) solution to afford metabolites. The metabolites were passed through a high-throughput tissue pulverizer Wonbio-96c (Shanghai Wanbo Biotechnology Co., Ltd., China), swirled, and ultrasonicated for 30 min. These were then centrifuged at 13,000 r for 15 min at 40°C, and the supernatant was taken in a sample bottle for the Ultra performance liquid chromatography/tandem mass spectrometry (LC-MS/MS) analysis.

The ropls (Version1.6.2, http://bioconductor.org/packages/release/bioc/html/ropls.html) R package from Bioconductor on the Majorbio Cloud Platform (https://cloud.majorbio.com) was used for the multivariate statistical analysis. Statistically significant groups were selected with variable importance in projection (VIP) values of >1 and *p* values of <0.05. Differential metabolites between the two groups were summarized and mapped into their biochemical pathways through metabolic enrichment and pathway analysis based on a database search (KEGG, http://www.genome.jp/kegg/) to obtain the biological pathway most relevant to experimental treatment.

### Statistical analysis

The data were expressed as the mean ± standard deviation (S.D.), and differences were considered significant when *P* was <0.05, as tested by one-way analysis of variance (ANOVA) using GraphPad Prism 8.0 (GraphPad Software, San Diego, CA, United States).

## Results

### SA deduced body weight and liver index in HFD-induced NASH rats

**FIGURE 2:**
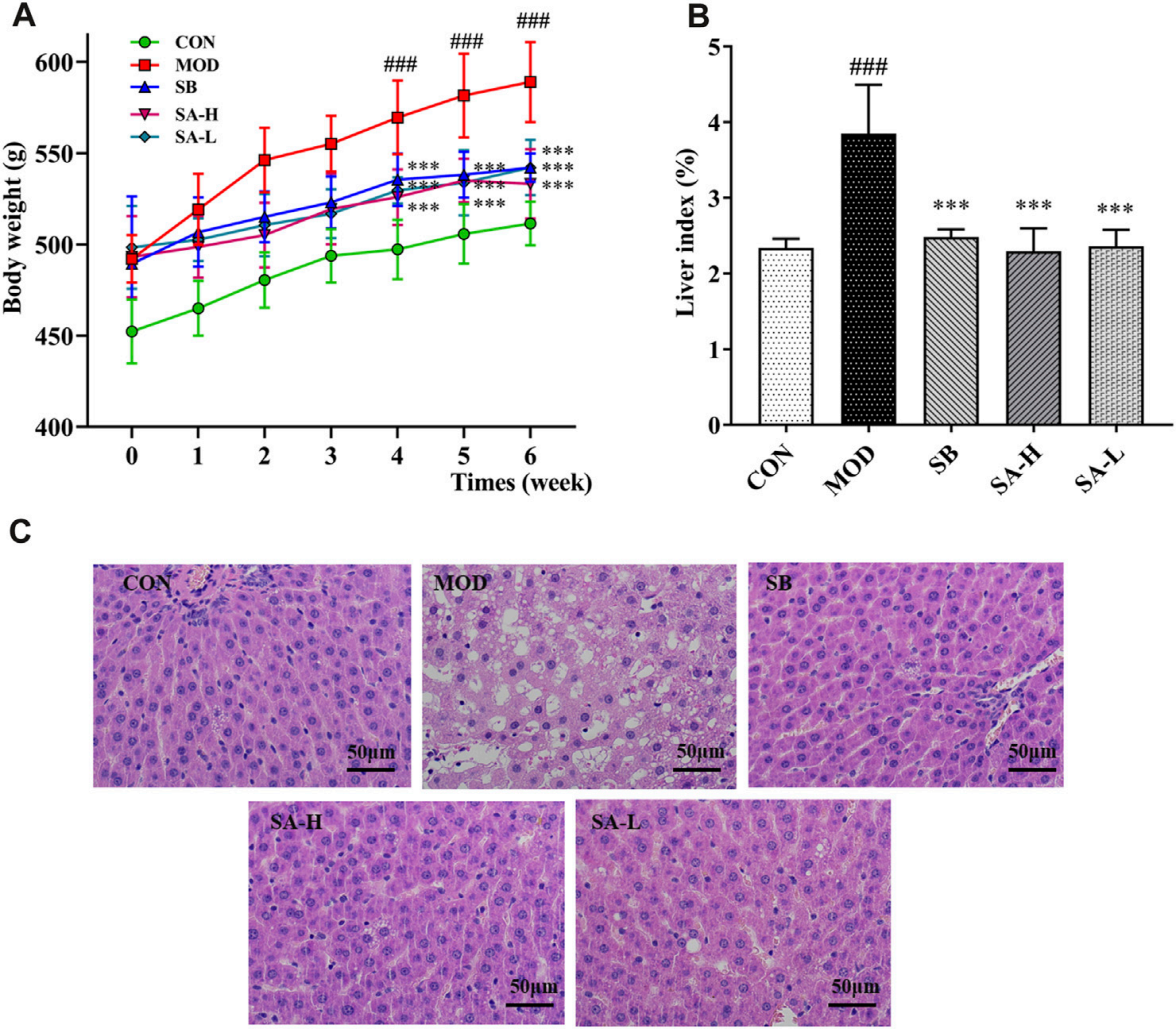
Phenotypic changes between the groups. **(A)** Changes in body weight after administration. **(B)** Liver index (%). **(C)** Hematoxylin and eosin staining of liver tissue. (×400, scale bar, 50 μm, *n* = 3). Differences were assessed by ANOVA. Data are expressed as the mean ± SD, *n* = 6 in each group. ^###^ *p* < 0.001 vs. the CON group and ^***^ *p* < 0.001 vs. the MOD group.

Because BW can increase in HFD-induced NASH rats, BW of NASH rats was calculated weekly to investigate the effects of SA, and BW of the CON and MOD groups steadily increased. BW of the MOD group was significantly increased compared with that of the CON group (*p* < 0.001). After 2 weeks of SB and SA administration, the BW of these groups slowly increased. At the end of the experiment, the BW of SB, SA-H, and SA-L groups was lower than that of the MOD group (*p* < 0.001) (Figure 2A). The liver index of the MOD group increased (*p* < 0.001), which was reversed by SA administration (Figure 2B). Liver pathological analysis showed that there was the blurred edge of liver cells, many vacuoles in the cytoplasm, big adipocyte, and severe hepatic steatosis in the MOD group. Compared with the MOD group, the SA group decreased hepatocyte vacuoles and liver volume, while significantly improving hepatic steatosis (Figure 2C). These results suggested that SA extract could reduce BW, inhibit hepatomegaly, and improve liver damage in HFD-induced NASH rats.

### SA regulated lipid metabolism, AST, and ALT in NASH rats

**FIGURE 3:**
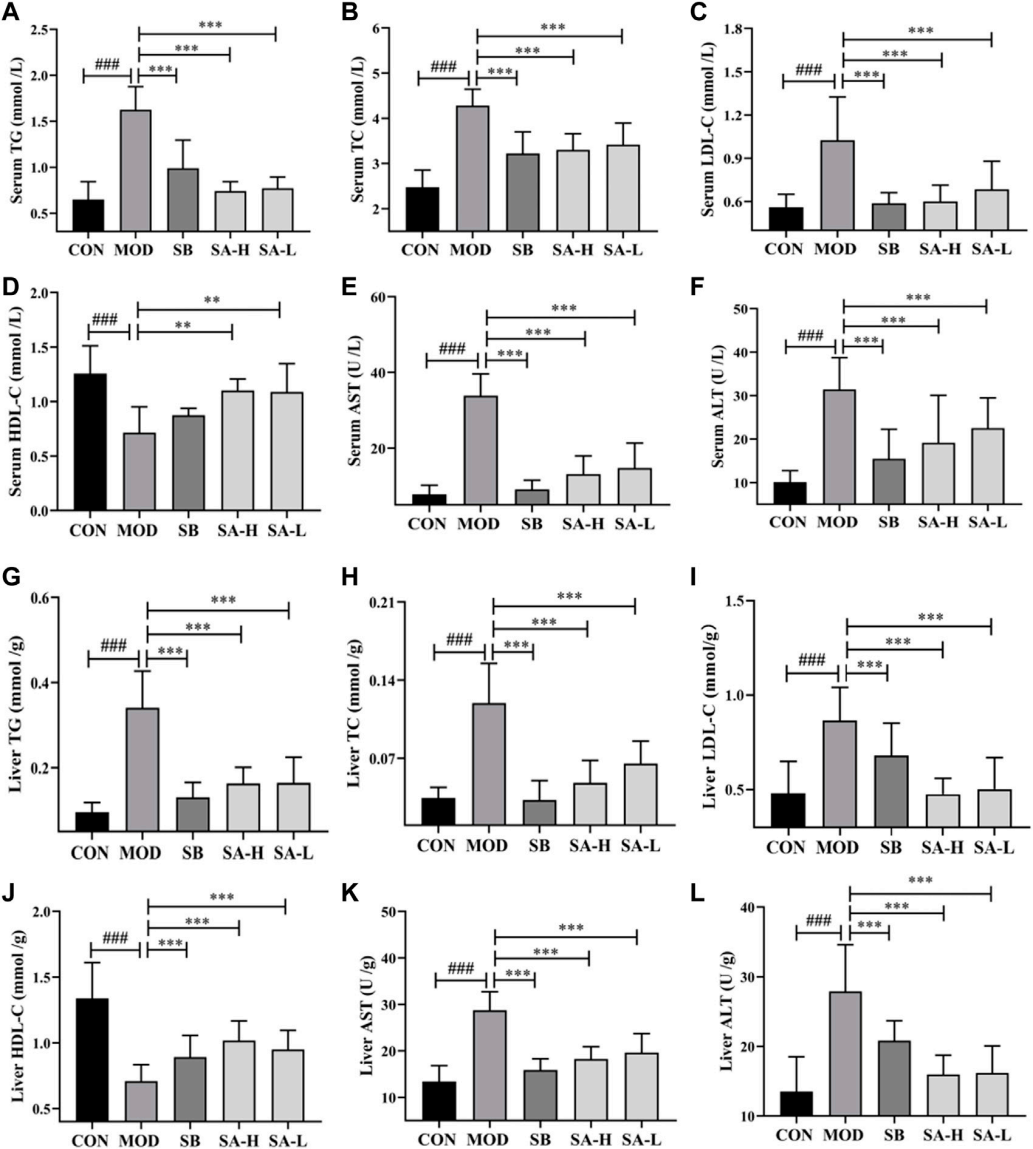
Indexes of serum and liver lipid metabolism and liver damage between the groups. **(A)** Serum TG. **(B)** Serum TC. **(C)** Serum LDL-C. **(D)** Serum HDL-C. **(E)** Serum AST. **(F)** Serum ALT. **(G)** Liver TG. **(H)** Liver TC. **(I)** Liver LDL-C. **(J)** Liver HDL-C. **(K)** Liver AST. **(L)** Liver ALT. Differences were assessed by ANOVA. Data are expressed as the mean ± SD, *n* = 6 in each group. ^###^ *p* < 0.001 vs. the CON control group and **p < 0.01, ^***^ *p* < 0.001 vs. the MOD group.

The liver is an important site of lipid metabolism, and lipid metabolism in NAFLD/NASH rats increases with the severity of the disease (Deprince et al., 2020). AST and ALT are important indexes to evaluate liver injury. Compared to the CON group, the levels of AST, TG, LDL-C, TC, and ALT (*p* < 0.001) in liver and serum significantly increased, while that of HDL-C (*p* < 0.001) significantly reduced compared to the NASH group. After gastric administration of SA, the levels of TG, AST, TC, ALT, and LDL-C (*p* < 0.001) in the liver and serum significantly decreased, while the level of HDL-C (*p* < 0.001) significantly increased compared to the NASH group (Figures 3A–L). These results indicated that SA could regulate lipid metabolism disorder and reduce liver injury in HFD-induced NASH rats.

### SA alleviated inflammatory cytokines in NASH rats

**FIGURE 4:**
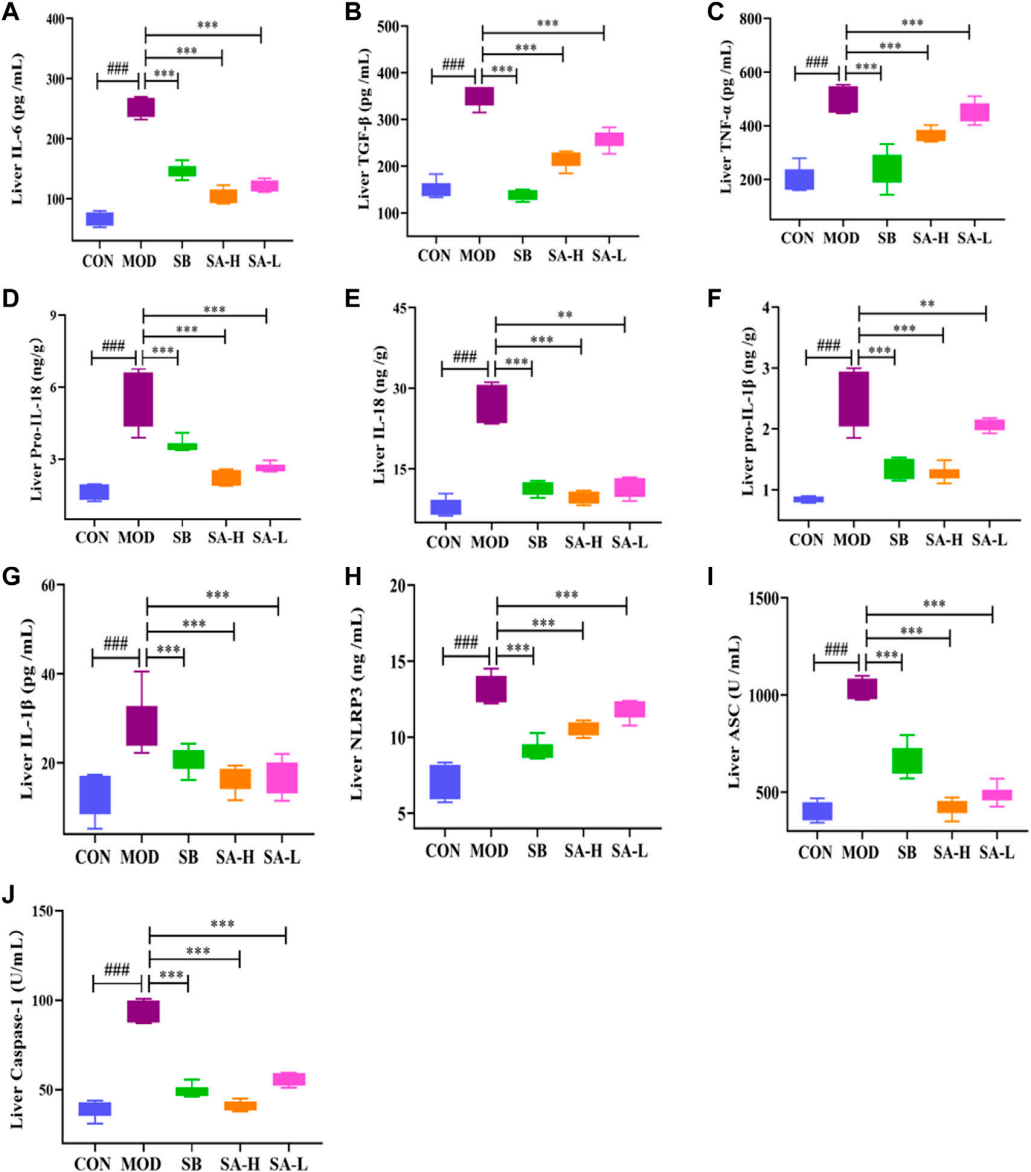
Liver inflammation levels between the group. **(A)** Liver IL-6. **(B)** Liver TGF-β. **(C)** Liver TNF-α. **(D)** Liver pro-IL-18. **(E)** Liver IL-18. **(F)** Liver pro-IL-1β. **(G)** Liver IL-1β. **(H)** Liver NLRP3. **(I)** Liver ASC. **(J)** Liver caspase-1. Differences were assessed by ANOVA. Data are expressed as the mean ± SD, *n* = 6 in each group. ^###^ *p* < 0.001 vs. the CON control group and ^**^ *p* < 0.01, ^***^ *p* < 0.001 vs. the MOD group.

NLRP3 plays a key role in the pathogenesis of NASH. Blocking activation of NLRP3 inflammasomes can reduce liver inflammation and improve NASH pathology (Xu et al., 2021). The inflammatory factors related to NLRP3 were detected by Elisa. The results showed that the levels of IL-18, pro-IL-18, pro-IL-1β, IL-1β, IL-6, TGF-β, TNF-α, ASC, caspase-1, and NLRP3 in the MOD group significantly increased (*p* < 0.001) compared with the CON group. However, SA relieved the increase levels of pro-IL-1β, IL-1β, NLRP3, IL-6, TGF-β, TNF-α, pro-IL-18, IL-18, caspase-1, and ASC (*p* < 0.001) in NASH rats (Figures 4A–J). These results suggested that SA could reduce the levels of inflammatory factors associated with NLRP3 inflammasome in the liver of HFD-induced NASH rats.

### SA inhibted NLRP3/ASC/caspase-1 axis in NASH

**FIGURE 5:**
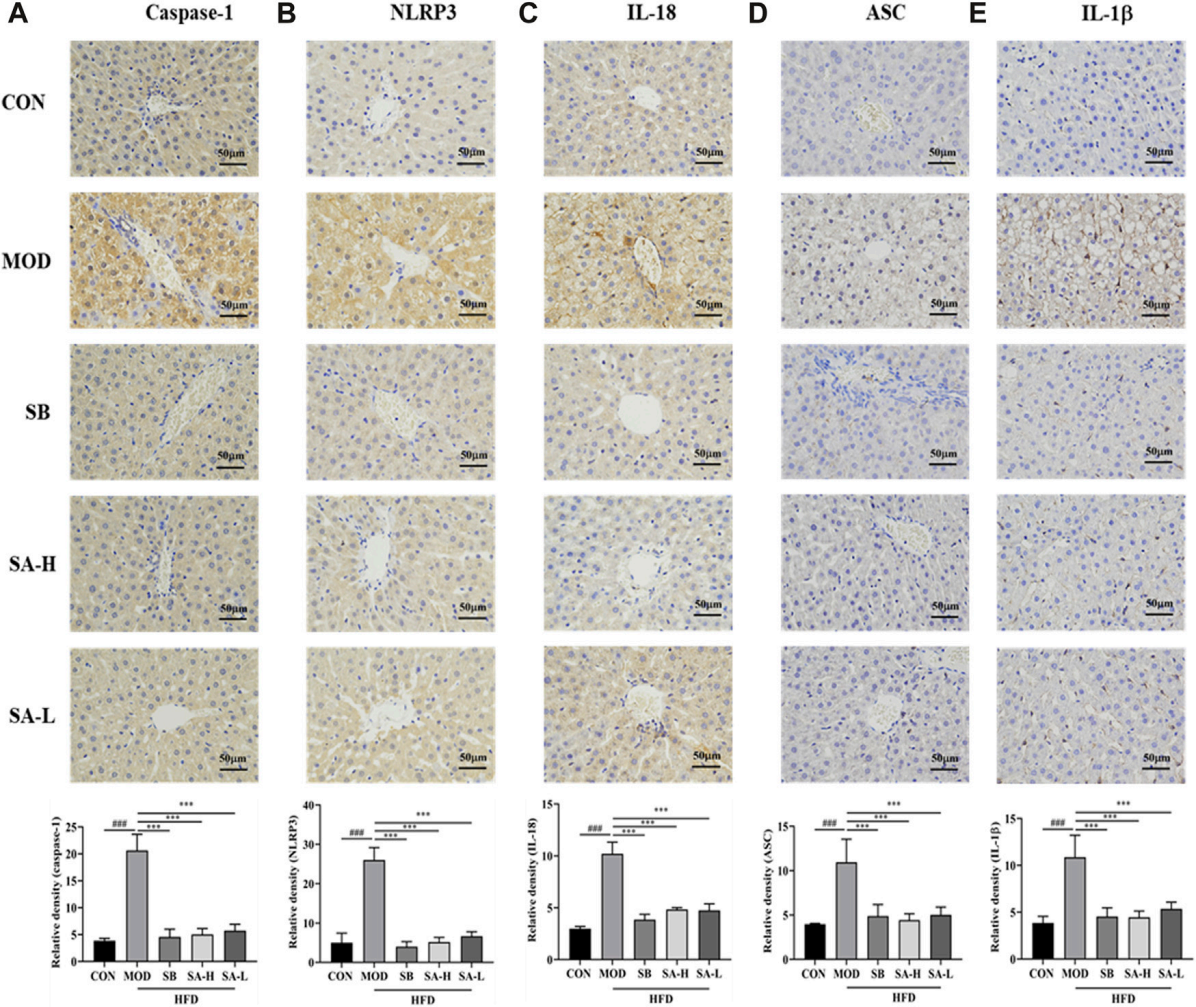
Immunohistochemical analysis of the effect of SA on the expression of ASC, caspase-1 and NLRP 3 proteins. **(A)** Expression level of Caspase-1. **(B)** Expression level of NLRP3. **(C)** Expression level of IL-18. **(D)** Expression level of ASC. **(E)** Expression level of IL-1β. Differences were assessed by ANOVA. Data are expressed as the mean ± SD, *n* = 3 in each group. ^###^ *p* < 0.001 vs. the CON control group and ^***^ *p* < 0.001 vs. the MOD group.

NASH rats were analyzed by immunohistochemistry to investigate the inhibitory effect of SA on NLRP3 inflammasomes (Figures 5A–E). Compared with the CON group, the positive expressions of caspase-1, IL-1β, IL-18, ASC, and NLRP3 in the MOD group significantly increased (*p* < 0.001). These effects were significantly reversed (*p* < 0.001) after treatment with the SA extract. Additionally, SA inhibited the increase of caspase-1, NLRP3, IL-18, ASC, and IL-1β levels, indicating that SA had a good inhibitory effect on the activation of NLRP3 inflammasomes.

**FIGURE 6:**
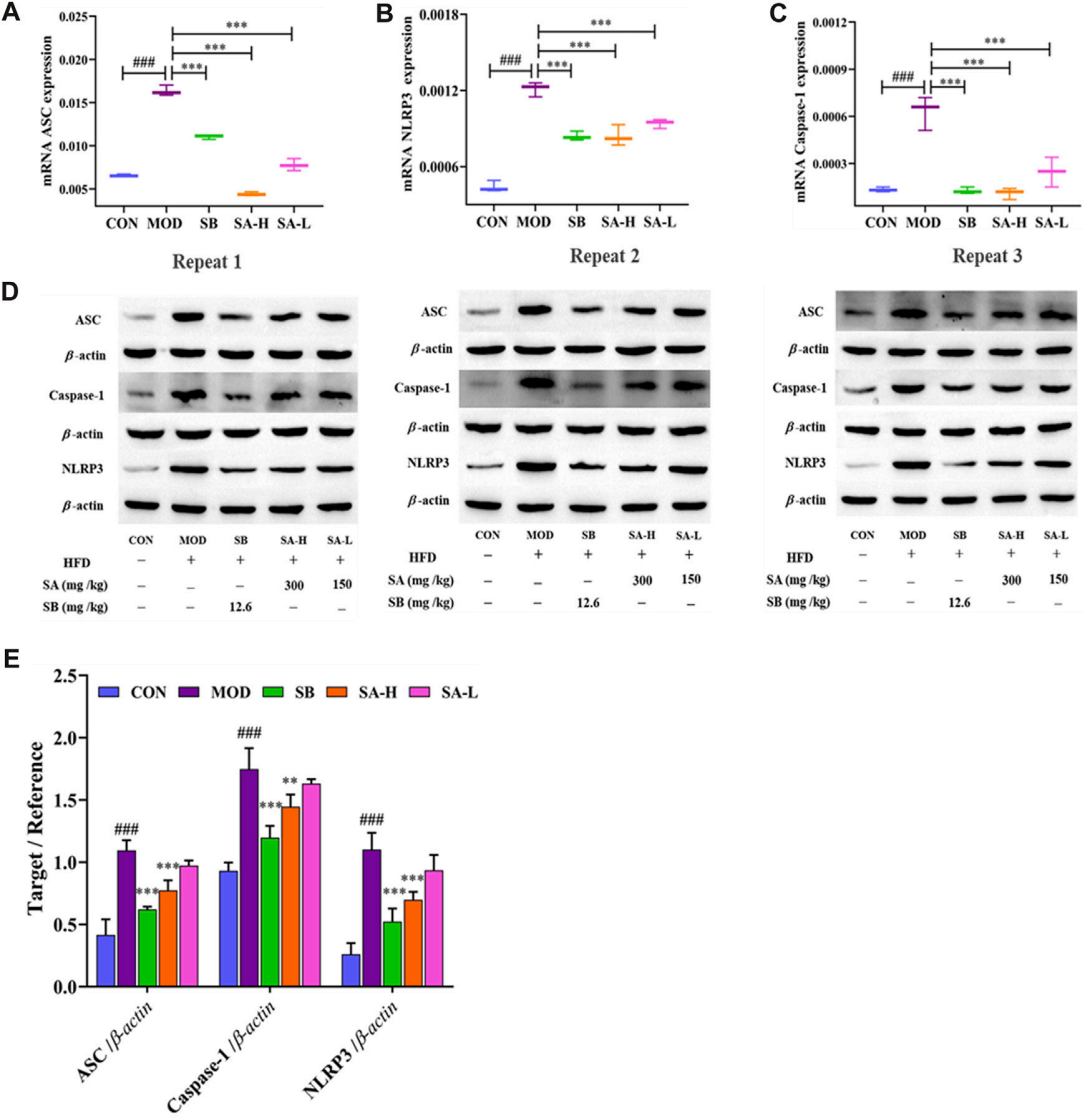
Effect of activation of NLRP3/ASC/caspase-1 inflammasomes signaling pathway on NASH. **(A–C)** mRNA levels of ASC, caspase-1, and NLRP3 were determined by RT-qPCR. **(D,E)** ASC, caspase-1, NLRP3 protein level in liver tissues of each group. Differences were assessed by ANOVA. Data are expressed as the mean ± SD, *n* = 3 in each group. ^###^ *p* < 0.001 vs. the CON control group and **p < 0.01, ^***^ *p* < 0.001 vs. the MOD group.

To further investigate the effect of NLRP3 activation on NASH, the expressions of NLRP3, ASC, and caspase-1 were measured by RT-qPCR (Figures 6A–C) and Western blot (Figures 6D, E) analysis. Interestingly, compared to the CON group, the expressions of NLRP3, ASC, and caspase-1 in the liver tissue of NASH rats were significantly increased. SA administration markedly suppressed the overexpression of NLRP3, ASC, and caspase-1. These results were consistent with immunohistochemical results. In summary, SA could inhibit the activation of NLRP3 in NASH rats to improve hepatomegaly, inflammation, and hepatic steatosis.

### SA recovered the disordered gut microbiome in HFD-induced NASH rats

16S rRNA gene sequencing was performed in each group of rats to explore the changes in the intestinal flora of NASH rats after SA administration and the effects of the changes in lipid metabolism and inflammatory factors associated with NLRP3 inflammasomes.

### Overall structure of gut microbiota

**FIGURE 7:**
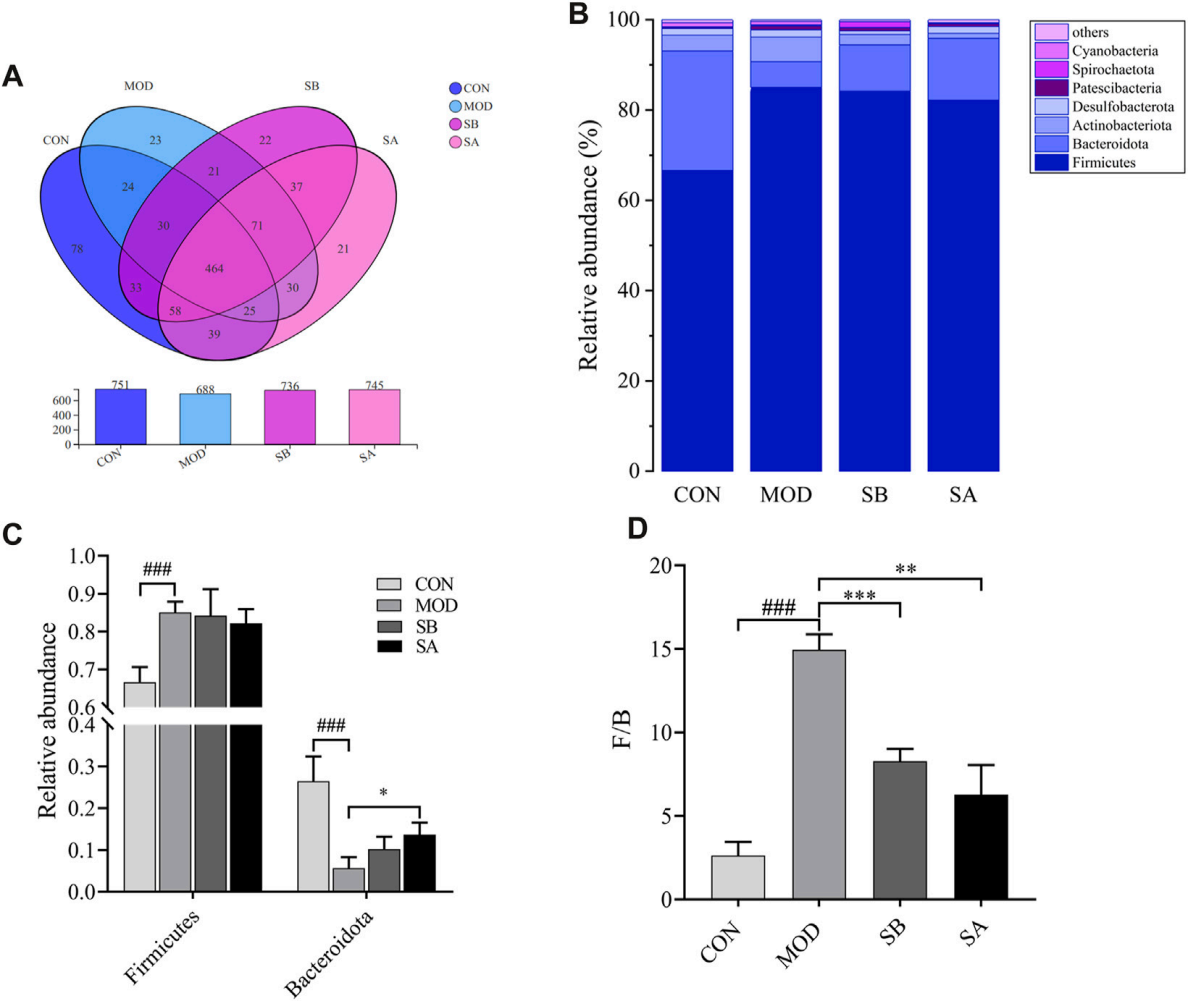
Venn diagram and the phylum level of gut microbiota in each group. **(A)** Venn diagram analysis at the phylum level. **(B)** Relative abundance of the intestinal flora at the phylum level. **(C)** Distribution of Firmicutes and Bacteroidetes at the phylum level. **(D)** Firmicutes/Bacteroidetes ratio. Differences were assessed by ANOVA. Data are expressed as the mean ± SD, *n* = 3 in each group. ^###^ *p* < 0.001 vs. the CON control group and ^**^ *p* < 0.01, ^***^ *p* < 0.001 vs. the MOD group.

The 16S rRNA gene sequencing identified a total of 976 operational taxonomic units (OTUs), 396 species, 222 genera, 108 families, 62 orders, 29 classes, 17 phyla, two kingdoms, and two domains. The similarity of OTUs among the CON, MOD, SB, and SA groups was shown by the Venn diagram. The similarity between the CON and SB groups was higher than that between the CON and MOD groups, while that between the CON and SA groups was higher than that between the CON and MOD groups. These results indicated more similarities in the intestinal microbiota composition between the SA and SB groups (Figure 7A).

### At the phylum level

At the phylum level (Figure 7B), compared with that in the CON group, the relative abundance of Firmicutes in the MOD group significantly increased (*p* < 0.001), while that in the SB and SA groups decreased. The relative abundance of Bacteroidota in the MOD group was significantly lower (*p* < 0.001) than that in the CON group, but that in the SB and SA groups significantly increased (*p* < 0.05) (Figure 7C). Notably, compared to that in the CON group, the Firmicutes/Bacteroidetes (F/B) ratio in the MOD group increased (*p* < 0.001). However, compared to that in the MOD group, the F/B ratio in the SB and SA groups significantly decreased (*p* < 0.001) (Figure 7D).

### At the family level

**FIGURE 8:**
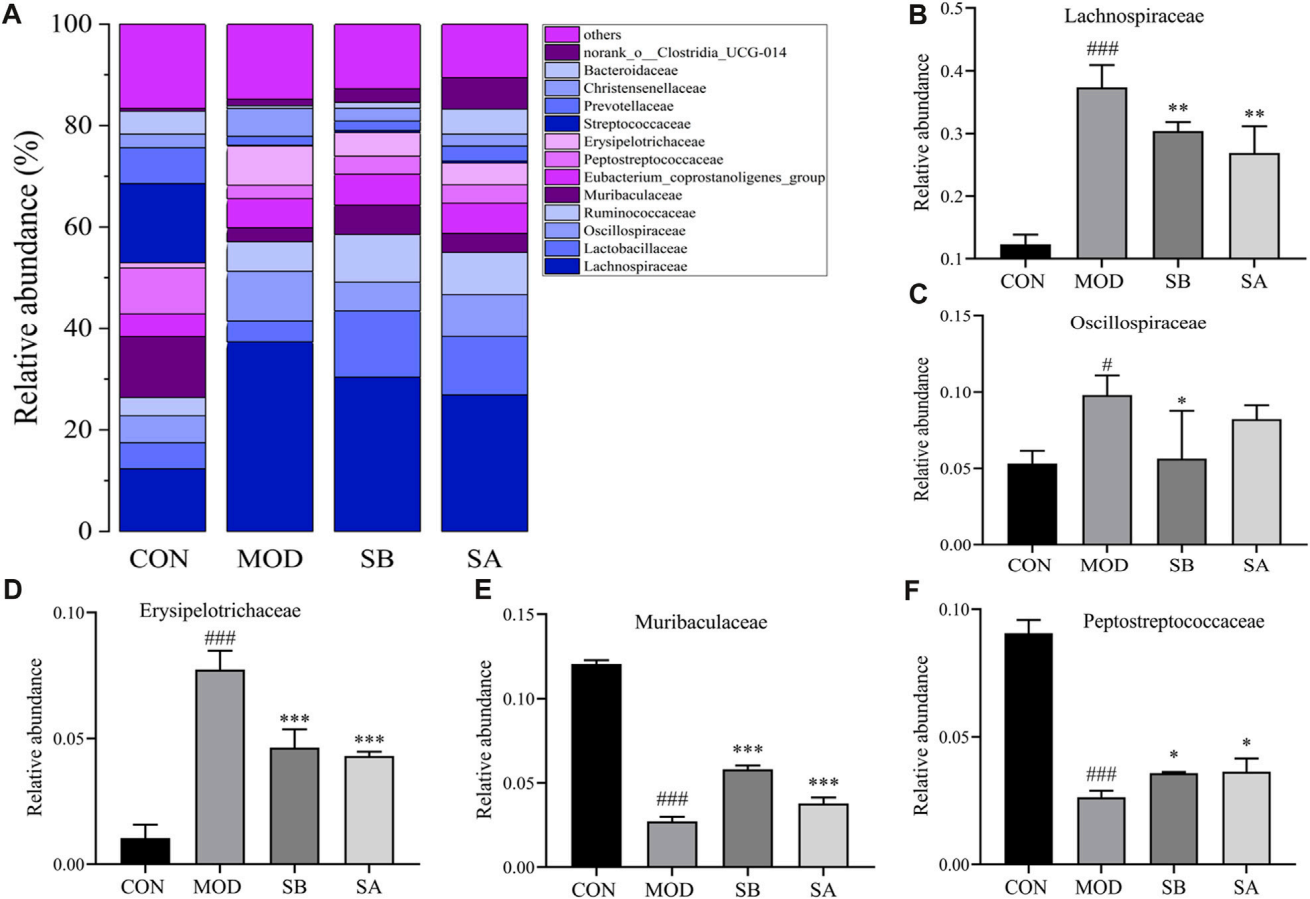
Gut microbiota at family classification level. **(A)** Relative abundance of the intestinal flora at the family level. **(B)** Lachnospiraceae. **(C)** Oscillospiraceae. **(D)** Erysipelotrichaceae. **(E)** Muribaculaceae. **(F)** Peptostreptococcaceae. Differences were assessed by ANOVA. Data are expressed as the mean ± SD, *n* = 3 in each group. ^###^ *p* < 0.00 vs. the CON control group and ^*^ *p* < 0.05, ^**^ *p* < 0.01, ^***^ *p* < 0.001 vs. the MOD group.

At the family level (Figure 8A), the relative abundances of Oscillospiraceae, Lachnospiraceae, and Erysipelotrichaceae in the MOD group significantly increased (*p* < 0.05, *p* < 0.001) compared to the CON group. As compared to the MOD group, the relative abundances of Erysipelotrichaceae (all *p* < 0.01), Oscillospiraceae (SB *p* < 0.05), and Lactobacillaceae (all *p* < 0.001) in the SB and SA groups were significantly reduced (Figures 8B–D). The relative abundances of Muribaculacae and Peptostreptococcaceae in the MOD group significantly decreased (*p* < 0.001) compared to the CON group. As compared to the MOD group, the relative abundances of Muribaculaceae (all *p* < 0.001) and Peptostreptococcaceae (all *p* < 0.05) in the SB and SA groups significantly increased (Figures 8E, F). These results indicated that SA could reverse the composition of intestinal flora in NASH rats.

### At the genus level

**FIGURE 9:**
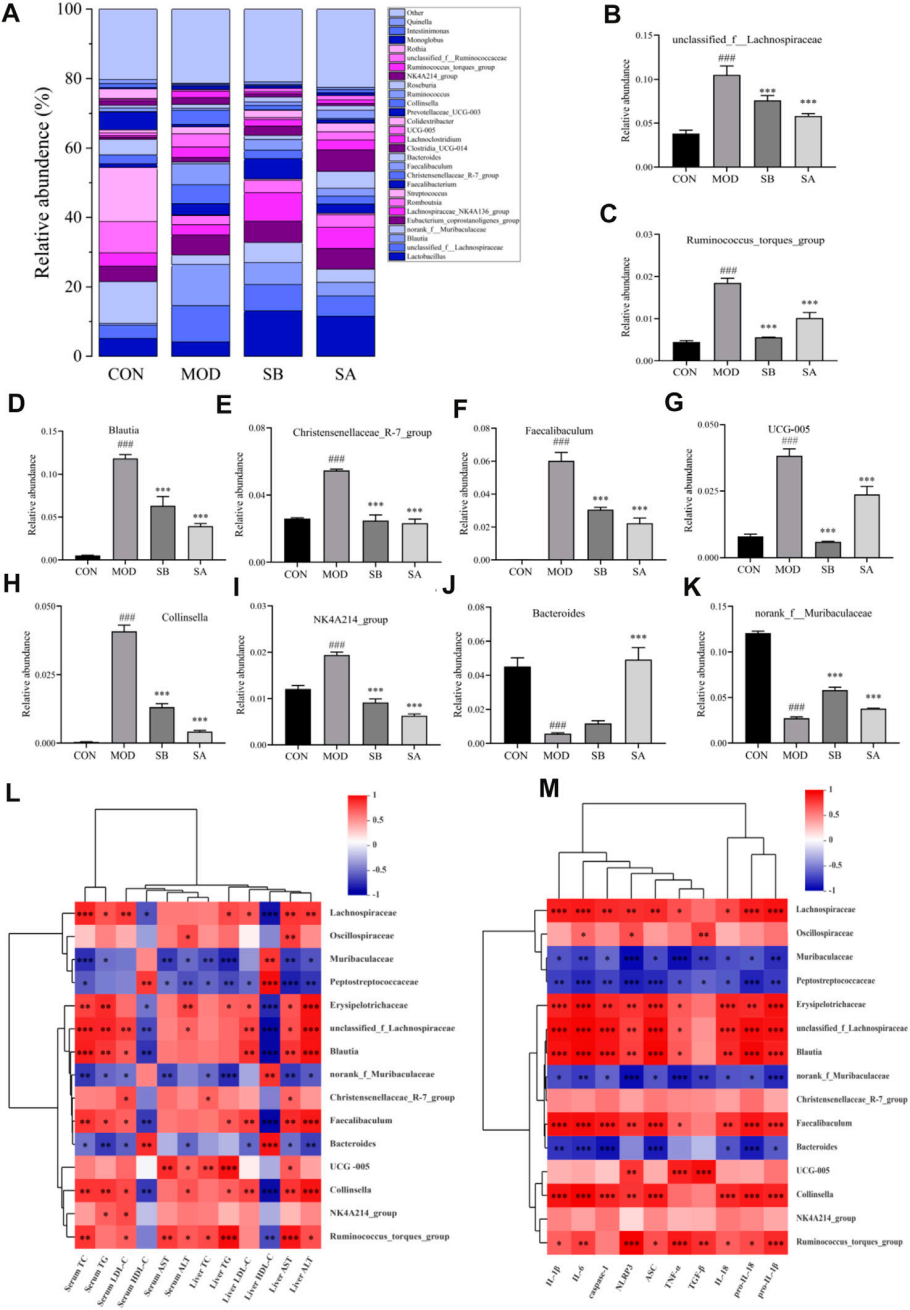
Gut microbiota at genus classification level and Spearman correlations between gut microbiota and lipid metabolism NLRP3-related inflammatory factors in each group. **(A)** Relative abundance of the intestinal flora at the genus level. **(B–K)** Distribution of gut microbiota at the genus level. **(L)** Gut microbiota and lipid metabolism. **(M)** Gut microbiota and NLRP3-related inflammatory factors. Differences were assessed by ANOVA. Data are expressed as the mean ± SD, *n* = 3 in each group. ^###^ *p* < 0.001 vs. the CON control group and ^*^ *p* < 0.05, ^**^ *p* < 0.01, ^***^ *p* < 0.001 vs. the MOD group.

At the genus level (Figure 9A), the abundances of *unclassified f* Lachnospiraceae, *Ruminococcus torques group*, *Blautia*, *UCG-005, Collinsella,* Christensenellaceae *R-7 group*, *Faecalibaculum*, and *NK4A214 group* in the MOD group were significantly increased (all *p* < 0.001) compared with the CON group. As compared to the MOD group, the relative abundances of these bacteria significantly decreased (all *p* < 0.001) in the SA and SB groups. Moreover, the relative abundances of *Bacteroides* (*p* < 0.001) and *norank_ f_Muribaculaceae* in the MOD group significantly decreased (all *p* < 0.001) compared with the CON group. The relative abundances of these microorganisms increased (all *p* < 0.001) in the SA and SB groups (Figures 9B–K) compared with the MOD group.

The Spearman correlation was analyzed to further determine the correlation between gut microbiota, lipid metabolism, and NLRP3-related inflammatory factors in each group. Spearman correlation analysis results showed that most of the intestinal flora was closely related to lipid metabolism, and NLRP3-related inflammatory factors (Figures 9L, M). The levels of TC, TG, LDL-C, AST, and ALT were positively correlated with the relative abundance of Lachnospiraceae (family), Oscillospiraceae (family), and *Blautia* (genus), but negatively correlated with the relative abundance of Muribaculaceae (family), and Peptostreptococcaceae (family). The improvement of NLRP3-related inflammatory factors was positively correlated with the relative abundance of Lachnospiraceae (family), Oscillospiraceae (family), and *Blautia* (genus). However, it was negatively correlated with the relative abundance of Muribaculaceae (family), Peptostreptococcaceae (family), *norank f Muribaculaceae* (genus), and *Bacteroides* (genus). Collectively, these results showed that SA improved lipid metabolism and reduced NLPR3-related inflammatory factors in NASH rats, which was closely related to the changes in gut microbiota.

### SA regulated the cecal contents metabolites in the HFD-induced NASH rats

We analyzed the key metabolites and lipid levels, NLRP3-related inflammatory factors, and intestinal microorganisms that change in NASH rats to investigate the effects of metabolite changes in NASH rats.

### Sample comparison analysis

**FIGURE 10:**
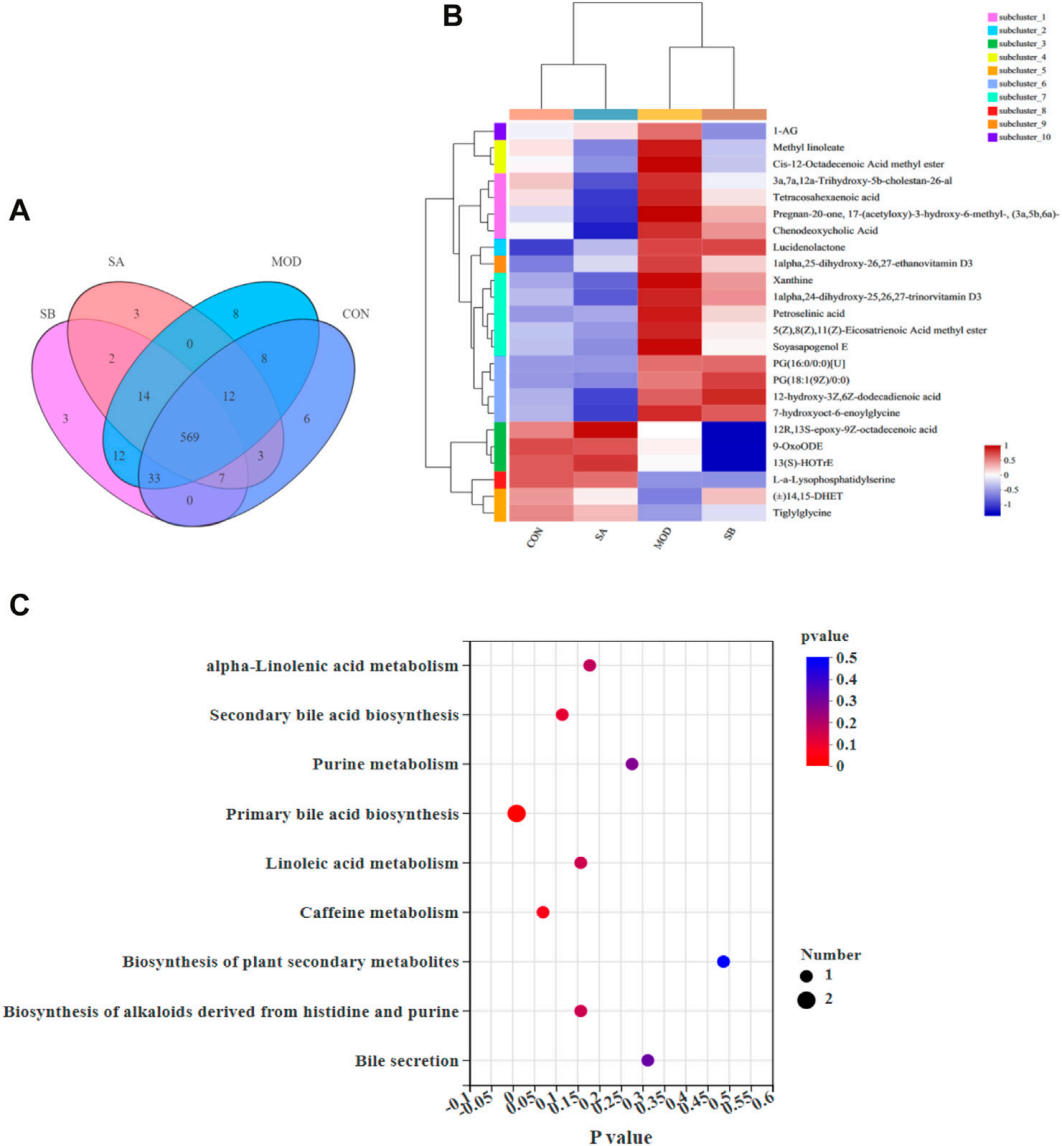
Sample comparison, differential metabolites and KEGG analysis. **(A)** Venn diagram, revealed the overlap of species in gut metabolites among the samples and number of metabolites in groups. **(B)** Analysis of dominant metabolites in CON, MOD, SB, and SA groups. **(C)** KEGG functional pathway analysis based on differential metabolites among the four groups.

The positive ion metabolic profiles of cecal contents samples were analyzed by UPLC-MS/MS. According to the Venn diagram, there are 569 metabolites in CON, MOD, SB, and SA groups (Figure 10A). Comparative analysis of different metabolites showed that the abundance levels of 24 metabolites were significantly reversed by SA. (*p*-value < 0.05) (Figure 10B). Most of the differential metabolites in the SA and SB groups were like those in the CON group. Interestingly, the SA group was close to the CON group, indicating that the SA had a significant ameliorating effect on NASH rats.

Twenty-four metabolites with significant differences are shown in Figure 8B, which was used to investigate the potential metabolic pathways of SA in NASH. The metabolic pathway analysis showed that SA regulated nine metabolic pathways in NASH rats (Figure 10C), including primary bile acid biosynthesis, alpha-linolenic acid metabolism, secondary bile acid biosynthesis, purine metabolism, linoleic acid metabolism, biosynthesis of alkaloids derived from histidine and purine, bile secretion. The metabolites mainly enriched in these metabolic pathways were 13(S)-HOTrE, xanthine, chenodeoxycholic acid (CDCA), 9-OxoODE, and 3a,7a,12a-trihydroxy-5b-cholestan-26-al (Table 1).

**TABLE 1:** Main metabolites enriched by nine metabolic pathways.

Metabolite pathway	Number	Name
alpha-linolenic acid metabolism	1	13(S)-HOTrE
Caffeine metabolism	1	Xanthine
Secondary bile acid biosynthesis	1	Chenodeoxycholic acid
Bile secretion	1	Chenodeoxycholic acid
Linoleic acid metabolism	1	9-OxoODE
Biosynthesis of alkaloids derived from histidine and purine	1	Xanthine
Purine metabolism	1	Xanthine
Biosynthesis of plant secondary metabolites	1	Xanthine
Primary bile acid biosynthesis	2	Chenodeoxycholic acid; 3a,7a,12a-trihydroxy-5b-cholestan-26-al

Metabolic pathways enriched by differential metabolites. The metabolites, which were enriched in 9 metabolic pathways, mainly included 13(S)-HOTrE, xanthine, chenodeoxycholic acid, 9-OxoODE, and 3a,7a,12a-trihydroxy-5b-cholestan-26-al.

The correlation between metabolites and lipid metabolism, inflammatory factors related to NLRP3, and gut microbes.

**FIGURE 11:**
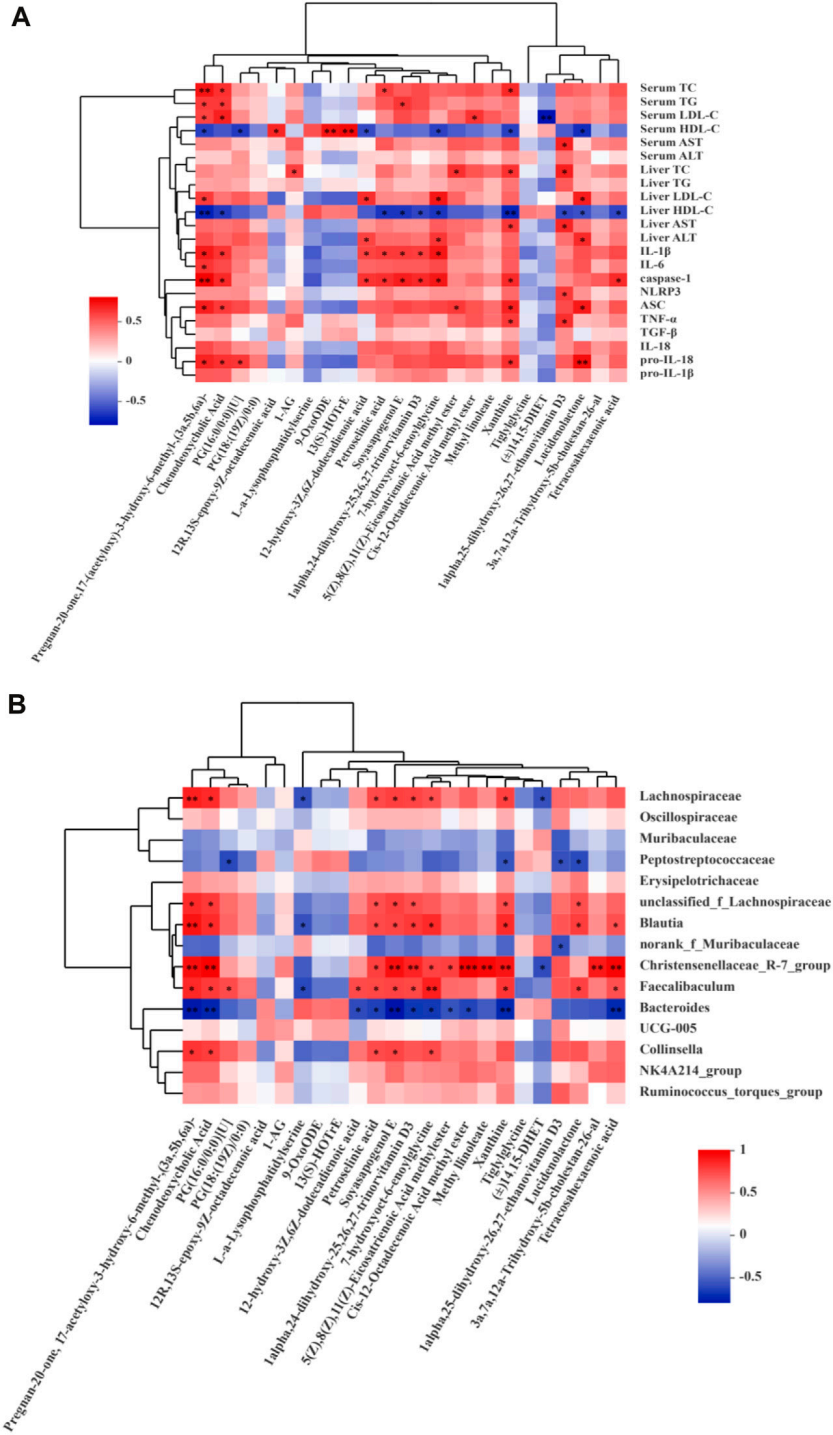
Spearman correlations analysis. **(A)** Metabolites and lipid metabolism, NLRP3-related inflammatory factors. **(B)** Metabolites and microbes at the family and genus levels in the CON, MOD, SB, SA groups of rats. Data are expressed as the mean ± SD, *n* = 3 in each group. ^*^ *p* < 0.05, ^**^ *p* < 0.01, ^***^ *p* < 0.001.

Spearman correlation analysis revealed that lipid metabolism improvement and NLRP3-related inflammatory factors were correlated (*p* < 0.05) with pregnan-20-one, 17-(acetyloxy)-3-hydroxy-6-methyl-, (3a,5b,6a)-, CDCA, 12-hydroxy-3z,6z-dodecadienoic acid, 7-hydroxyoct-6-enoylglycine, xanthine, 1-alpha,25-dihydroxy-26,27-ethanovitamin D3, and lucidenolactone (Figure 11A). Meanwhile, these metabolites were also significantly correlated with Lachnospiraceae (family), *Blautia* (genus), Christensenellaceae *R-7 group* (genus), *Faecalibaculum* (genus), *Bacteroides* (genus), and *Collinsella* (genus) (Figure 11B).

## Discussion

NASH is a chronic metabolic disease characterized by fatty liver, hepatocyte injury, and inflammation (Povsic et al., 2019). According to Yunnan National Medicine Dictionary, SA is bitter in taste, cold in nature, and belongs to the liver, large intestine meridians, lungs, and stomach, which is often used to treat liver pain, infectious hepatitis, lung cough, and other diseases in Yi medicine (Wang et al., 2022). Traditional Chinese medicine (TCM) is characterized by personalized, multi-target, multi-component, and holistic treatment strategies. A growing number of studies have found that NLRP3 may be an important target for the treatment of NAFLD/NASH (Wan et al., 2016; Thomas, 2017). This study investigated the regulatory effects of SA on NLPR3 inflammatory factors in NASH rats and its mechanism, while metabolomics and 16S rRNA gene sequencing were used to elucidate the mechanism of SA in NASH. The study showed that SA had therapeutic effects in NASH rats and the levels of ALT, TG, AST, LDL-C, and TC in rat serum and liver decreased (*p* < 0.001), while that of HDL-C increased (*p* < 0.001). These results are consistent with those of Hwangbo et al. (2020) and Zhou et al. (2021), and NLRP3-related inflammatory factors, such as caspase-1, pro-IL-18, TNF-α, IL-18, pro-IL-1β, NLRP3, IL-1β, ASC, IL-6, and TGF-β significantly increased (*p* < 0.001) in NASH rats by HFD-induced. These effects were restored by the SA extract and SB treatment, and these were consistent with the results of Dong et al. (2020) and Shi et al. (2021). These changes may also be related to the activity of SA, increasing the abundance of beneficial microflora, and reducing the growth of harmful microflora (Wang et al., 2019). Interestingly, we found that SA also could regulate the balance of gut microbiota and its metabolism disorder in NASH rats by inhibiting NLRP3/ASC/caspase-1 axis.

Some studies have shown that an intricate balance exists between the gut microbiome, diet, and NLRP3 function. The pathogenesis of NASH comprises multiple steps of interaction between intestinal microbiota and the host, which might be the cause of derangement in lipid metabolism, leading to metabolic diseases (Pierantonelli et al., 2017). Activation of inflammasome components has been shown in patients with chronic liver injury, particularly NAFLD/NASH, and an NLRP3 selective inhibitor improved fibrosis in obese diabetic and NAFLD pathology mice (Sellin et al., 2015; Mridha et al., 2017). Therefore, NLRP3-inflammasome is essential for maintaining intestinal homeostasis. At the phylum level, the abundance of Bacteroidetes significantly decreased (*p* < 0.001), while that of Firmicutes significantly increased (*p* < 0.001), and the F/B ratio significantly increased (*p* < 0.001) in the MOD group. The F/B ratio significantly reduced (*p* < 0.001) after SA administration, which was consistent with the results of other studies demonstrating that the reduction of F/B ratio could effectively reduce obesity (Zhang et al., 2021). At the family level, Lachnospiraceae is a crucial family of bacteria in human intestinal microbiota, which is short-chain fatty acids (SCFAs)-producing bacteria (Chen et al., 2018). The production of SCFAs leads to a decrease in intestinal permeability, leading to many harmful substances, such as LPS, and inflammatory factors entering the blood and causing hepatotoxicity (Sun et al., 2022). At the genus level, *R. torques group* and *Blautia* levels have a positive relationship to lipid deposition in NASH rats, mainly producing butyrate, which can promote lipid deposition (Boursier et al., 2016; Lyu et al., 2021). SA also reversed HFD-induced significant changes in the abundance of certain genera, including *Christensenellaceaeb R-7 group* (genus), *Faecalibaculum* (genus), *UCG-005* (genus), *Collinsella* (genus), *NK4A214 group* (genus), *Bacteroides* (genus), and *norank f Muribaculaceae* (genus). This experiment showed that SA could reduce liver injury and alleviate the increase in inflammatory factors by reversing the changes in these intestinal microbiotas.

In addition to the imbalance of intestinal flora composition in HFD-induced NASH rats, some metabolites produced by commensal bacteria may also promote the progression of NASH. However, there are few studies on metabolic disorders of NASH. In this study, we found that SA and SB could reverse 24 metabolites, and the effect of SA was more significant than that of SB. Nine main metabolic pathways were found, including linoleic acid metabolism, alpha-linolenic acid metabolism, secondary bile acid biosynthesis, purine metabolism, biosynthesis of alkaloids derived from histidine and purine, primary bile acid biosynthesis, and bile secretion. These metabolic pathways may play an important role in SA improving NASH, among them, the secondary bile acid biosynthesis is closely related to the flora of Lachnospiraceae (family) and Ruminococcaceae (family). It also is associated with several diseases such as liver cirrhosis, enteritis, and cancer (Sinha et al., 2020; Guzior and Quinn, 2021). Purine metabolism is closely related to the intestinal content of *Lactobacillus* (family). Excessive Purine metabolites may cause uric acid too high and stimulate the increase of various inflammatory factors, such as TNF-α, IL-6, and IL-1β (Wu et al., 2020). The metabolites mainly enriched in these metabolic pathways were 13(S)-HOTrE, xanthine, CDCA, 9-OxoODE, and 3a,7a,12a-Trihydroxy-5b-cholestan-26-al. These metabolic pathways and metabolites may be the key metabolic pathways and metabolites for SA to improve NASH. 13(S)-HOTrE is a key metabolite that can inactivate NLRP3 and downregulate LPS-induced inflammatory markers (Kumar et al., 2016). Excessive xanthin can cause hyperuricemia and then lead to NAFLD and NASH (Nishikawa et al., 2020). CDCA can be converted into secondary bile acids, such as deoxycholic acid (DCA), and regulate intestinal flora including *Faecalibacterium*, *Roseburia*, and *Lachnospira*, thus, interfering with NASH (Jiao et al., 2018; Lee et al., 2023). In our experiment, SA can maintain the metabolic balance by regulating metabolites such as xanthine, CDCA, and 3a,7a,12a-Trihydroxy-5b-cholestan-26-al etc. In sum, the mechanism of action of key metabolites regulated by SA needs further investigation. The metabolic pathways, such as secondary bile acid biosynthesis, alpha-linolenic acid metabolism, and purine metabolism, may play an irreplaceable role in SA improving the metabolic disorder of NASH rats.

Correlation analysis showed that most intestinal microflora components were closely related to lipid metabolism and NLRP3-related inflammatory factors. The levels of Muribaculaceae (family), Peptostreptococcaceae (family), *norank f Muribaculaceae* (genus), and *Bacteroides* (genus) were negatively correlated with TC, TG, LDL-C, AST, ALT, IL-6, TGF-β, TNF-α, pro-IL-18, IL-18, pro-IL-1β, IL-1β, NLRP3, ASC, and caspase-1 but positively correlated with HDL-C. Lachnospiraceae (family), Oscillospiraceae (family), Erysipelotrichaceae (family), *Blautia* (genus)*, Christensenellaceaeb R-7 group* (genus), *Faecalibaculum* (genus), *UCG-005* (genus), *Collinsella* (genus), *NK4A214 group* (genus), *R. torques group* (genus) had the opposite correlation with them. Additionally, some metabolites, such as CDCA, pregnan-20-one, 17-(acetyloxy)-3-hydroxy-6-methyl-, (3a,5b,6a)-, 12-hydroxy-3z,6z-dodecadienoic acid, 7-hydroxyoct-6-enoylglycine, xanthine, 1-alpha,25-dihydroxy-26,27-ethanovitamin D3, and lucidenolactone, were positively correlated with AST, TC, ALT, LDL-C, TG, and NLRP3-related inflammatory factors. Interestingly, these metabolites also had a strong correlation with intestinal flora components, such as Lachnospiraceae (family), *Blautia* (genus), Christensenellaceae *R-7 group* (genus), *Faecalibaculum* (genus), and *Bacteroides* (genus). CDCA In this study, these intestinal florae and their metabolites may be closely related to the activation of NLRP3/ASC/caspase-1 axis. The relationship between these florae and their metabolites and NLRP3/ASC/caspase-1 axis can be further studied, and the mechanism of SA improving NASH can be more clearly defined.

Taken together, our results show that SA treatment can protect against HFD-induced BW gain and regulate lipid metabolism, liver steatosis, inflammation, intestinal flora imbalance, and metabolic disorders. This may be associated with the inhibition of the NLRP3/ASC/caspase-1 axis activation caused by NASH. Notably, in this study, SA improved NASH by inhibiting NLRP3. Moreover, some intestinal florae, such as Lachnospiraceae (family), *Blautia* (genus), Christensenellaceae *R-7 group* (genus), *Faecalibaculum* (genus), and metabolites, including CDCA, 12-hydroxy-3z,6z-dodecadienoic acid, pregnan-20-one, 17-(acetyloxy)-3-hydroxy-6-methyl-, (3a,5b,6a)-, 7-hydroxyoct-6-enoylglycine, xanthine, 1-alpha,25-dihydroxy-26,27-ethanovitamin D3, and lucidenolactone, also had a strong correlation with NLRP3. Therefore, SA can improve NASH by regulating the NLRP3/ASC/caspase-1axis, its related intestinal flora composition, and metabolic disorder. This study provides useful hints for the treatment of NASH and is helpful for the utilization of SA resources and the development of new therapeutic drugs.

## Conclusion

This study showed that SA could reduce the weight gain of HFD-induced NASH rats and the liver index. SA could also regulate lipid metabolism disorder and reduce inflammation in NASH rats. Most importantly, we found that SA significantly inhibited the activation of NLRP3 and regulated the NLRP3/ASC/caspase-1 axis. Particularly, SA could obviously improve intestinal flora composition and metabolic disorder, and prevent HFD-induced NASH in rats, and nine main metabolic pathways were found, such as purine metabolism, alpha-linolenic acid metabolism, primary bile acid biosynthesis, secondary bile acid biosynthesis, Caffeine metabolism, linoleic acid metabolism, biosynthesis of alkaloids derived from histidine and purine, biosynthesis of plant secondary metabolites, bile secretion. In addition, some intestinal florae, such as Lachnospiraceae (family), *Blautia* (genus), and Christensenellaceae *R-7 group* (genus), and metabolites, such as CDCA, xanthine, 13(S)-HOTrE, 9-OxoODE, were closely related to the regulation of the NLRP3/ASC/caspase-1 axis by SA, and these findings provide a new research direction for the prevention and treatment of NASH.

## Data Availability

The original contributions presented in the study are publicly available. This data can be found here: https://doi.org/10.6084/m9.figshare.24428092.
